# Pulmonary Barotrauma After Diving Without Breathing Equipment

**DOI:** 10.7759/cureus.47382

**Published:** 2023-10-20

**Authors:** Corinne Rezentes, Chavez Scott

**Affiliations:** 1 Emergency Medicine, Brooke Army Medical Center, San Antonio, USA

**Keywords:** airway management, subcutaneous emphysema, soft tissue crepitus, diving related illness, pulmonary barotrauma

## Abstract

A case of a 19-year-old male with mediastinal and subcutaneous emphysema consistent with pulmonary barotrauma after diving is reported. He presented with facial swelling, voice change, chest pain, and shortness of breath after multiple dives between 8 and 12 feet deep without underwater breathing equipment in a river. Relevant radiology, including radiographs and computed tomography (CT imaging), and a discussion of pulmonary barotrauma are presented.

## Introduction

Barotrauma is defined as physical damage to the body tissues due to a pressure difference between the body and the surrounding environment. Pulmonary barotrauma refers to the pulmonary tissues and the damage to those tissues due to the difference in pressure. Commonly pulmonary barotrauma occurs iatrogenically in the setting of high-pressure invasive ventilation. Existing literature regarding barotrauma from diving is typically related to sinus or middle ear trauma and less frequently pulmonary. This case discusses a potential etiology of pulmonary barotrauma in the setting of underwater diving, in this case diving underwater into a higher pressured environment without any supplemental breathing equipment, and the importance of quick identification and intervention in the emergency department.

## Case presentation

A 19-year-old previously healthy male, a nonsmoker with no pulmonary history presented with voice change, face and neck swelling, chest pain, and shortness of breath after diving in a river. The patient reported diving approximately 10 to 12 feet without using a mask or any additional diving gear, including oxygen tanks or breathing apparatuses. After a couple of dives, and no known trauma during these dives, his friends pointed out that his voice sounded different. Within the next two hours, he developed facial swelling along with chest pain and shortness of breath. The patient denied aspirating any water during the dives or rapidly ascending. Emergency medical services were called and gave him 0.3 milligrams of intramuscular epinephrine and 50 milligrams of intravenous diphenhydramine due to fear this could be anaphylaxis; however, there was minimal improvement with these interventions. Upon arrival at the emergency department, the patient was hemodynamically stable, with initial vitals of heart rate of 66 beats per minute, respiratory rate of 18 per minute, blood pressure of 115/61, and oxygen saturation of 98% on room air.

Physical exam was significant for swelling over the neck and shoulders with underlying palpable crepitus, lungs clear to auscultation bilaterally with equal chest wall excursion, regular heart rate and rhythm, head atraumatic and normocephalic, neck atraumatic, edematous oropharynx extending to the posterior pharynx, and lip and tongue swelling. The patient had a muffled voice noted with tonsillar pillars touching during phonation and did not have any rashes or neurologic deficits.

**Figure 1:**
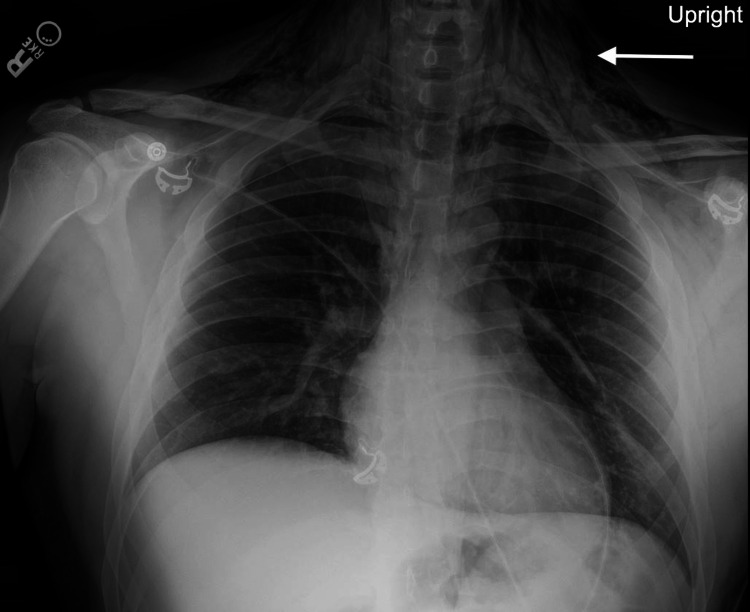
Initial chest radiograph with an arrow pointing to subcutaneous air in the neck

**Figure 2:**
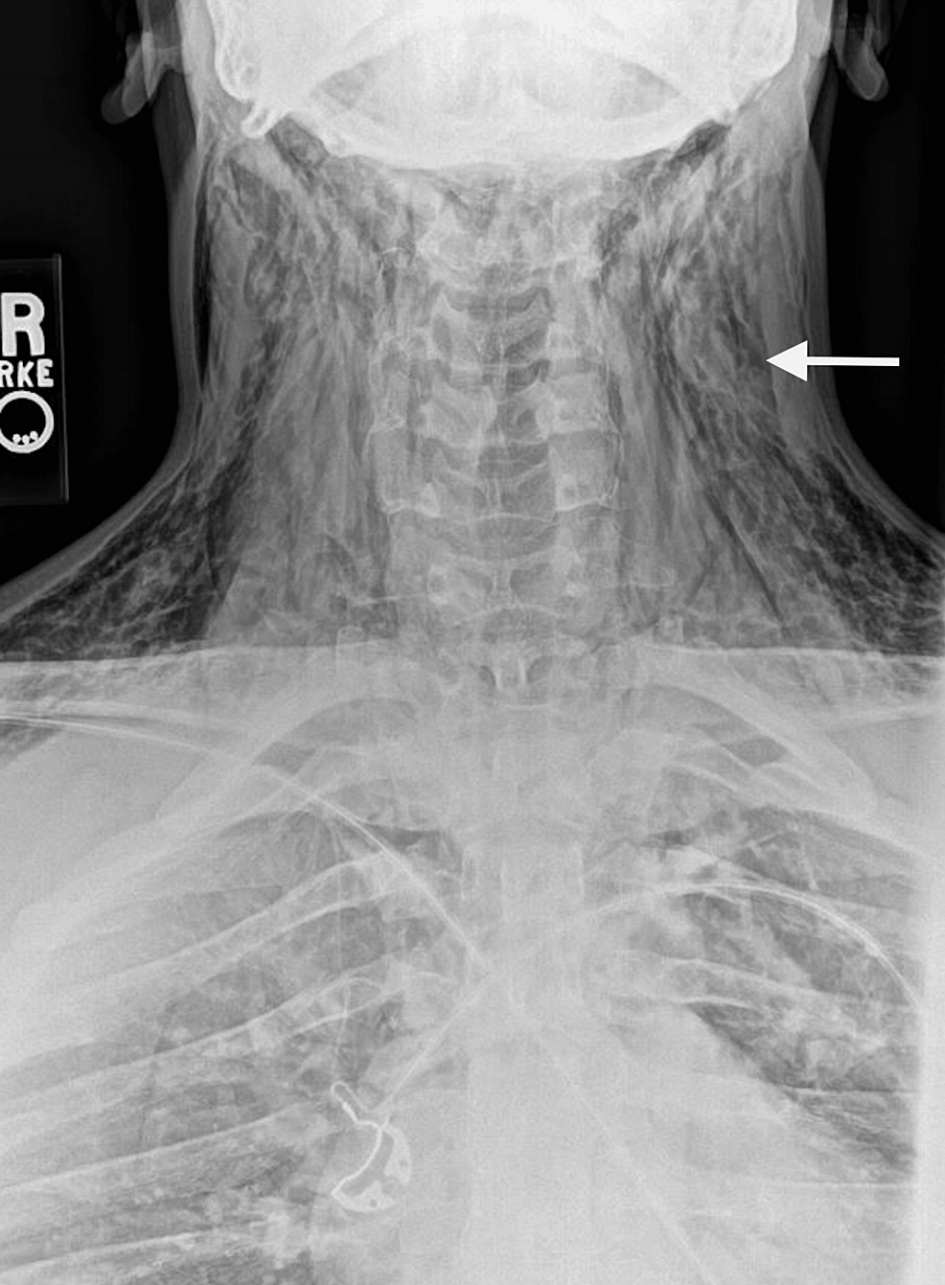
Neck radiograph with an arrow pointing to subcutaneous air

Initial interventions included 12 milligrams of intravenous dexamethasone for the significant oropharynx and airway edema. Chest radiograph revealed large volume mediastinal and neck emphysema and concern for possible aspiration in the bilateral lower lobes (Figure 1). The neck radiograph showed severe subcutaneous emphysema (Figure 2).

Given the progressive nature of his presentation and concern for airway compromise, the patient and provider used shared decision-making to opt to secure the patient’s airway. The patient was intubated using 160 milligrams of ketamine, 80 milligrams of rocuronium, and 7.5 endotracheal tube (ETT) and then was subsequently ventilated and sedated with propofol and ketamine. Initial ventilator settings were lung protective given unclear pathology at this point. Settings included a volume control mode with a tidal volume of 420 ml, positive expiratory end pressure of 5 mmHg, fraction of inspired oxygen of 100%, and peak pressure of 18 mmHg.

**Figure 3:**
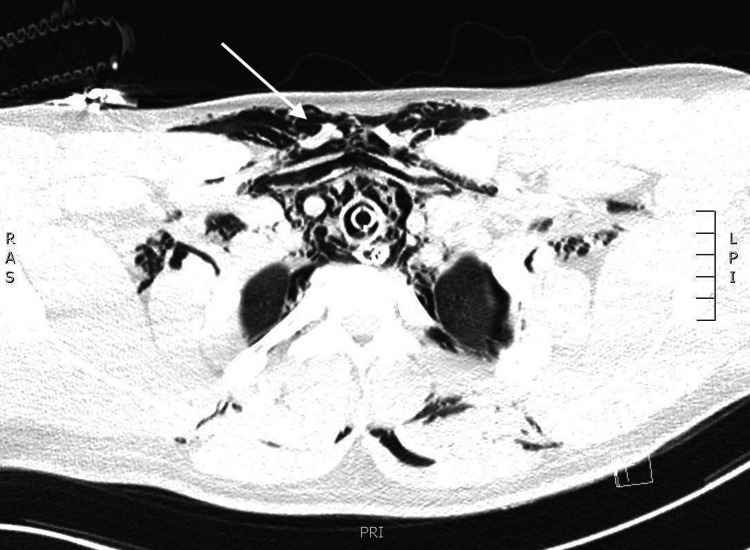
Chest CT highlighting the mediastinal air

The patient's computed tomography (CT) of his chest revealed fluid filling of the bilateral lower lobe bronchi and diffuse interstitial emphysema throughout both lungs suggesting that the patient’s mediastinal and subcutaneous emphysema was secondary to diffusely increased alveolar pressures which can occur with asthma, coughing, and barotrauma (Figure 3).

**Figure 4:**
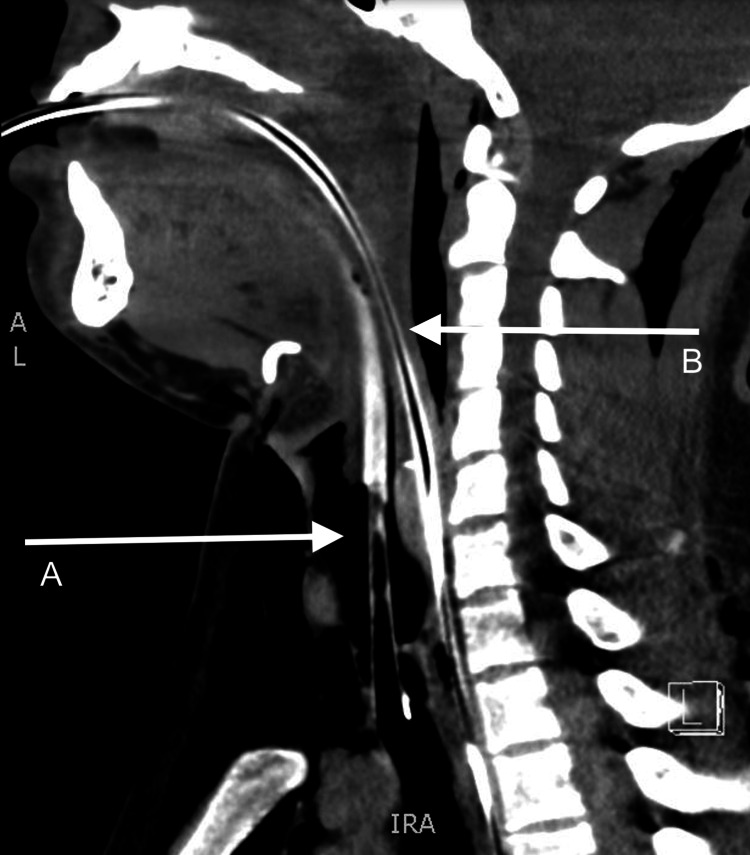
CT of the neck with arrow A pointing to an intact esophagus and arrow B pointing to ETT in the intact trachea ETT: endotracheal tube

CT of his neck showed extensive emphysema of the neck with visualized intact portions of the esophagus, pharynx, larynx, and trachea without obvious injury or defect (Figure 4). No significant worsening of the emphysema was noted on imaging after the intubation.

The patient ultimately was admitted to the intensive care unit for further evaluation and management for what was likely pulmonary barotrauma secondary to the repetitive dives. The patient was successfully extubated the following day. Subcutaneous emphysema continued to be present but was improving. The patient was evaluated by pulmonology in the hospital who recommended outpatient evaluation to include pulmonary function tests and instruction to not go diving anymore.

## Discussion

This case is unique given the patient’s lack of lung disease history and the arguably mild to moderate depths at which he dove without any underwater breathing equipment, specifically without compressed air. Barotrauma is defined as physical damage to the body tissues due to a pressure difference between the body and surrounding environment, fluid in the case of diving, and air in the case of flying [1,2]. Boyle’s law states that as pressure decreases the volume of a gas must increase and the opposite must be true as well, as the pressure increases the volume of a gas must decrease. When a diver ascends the pressure the body is experiencing decreases, and thus the volume of gas in the lungs increases and must be exhaled [3]. In the case of barotrauma as the gas in the lung expands, if the diver does not exhale, this increased gas volume in the lungs can cause damage [3]. What makes this case unique is the lack of compressed air utilization. This patient inhaled a set volume of air, dove to a certain depth, and returned to the surface without increasing the lung gas volume past the original volume, yet sustained obvious barotrauma manifesting as mediastinal and subcutaneous emphysema [4]. Additional considerations include any aspiration, coughing, or potential laryngospasm that could have caused an acute increase in pressure and thus the barotrauma. Clinical signs of this trauma can include subcutaneous emphysema, hypoxemia, hypotension, tachycardia, unilateral breath sounds, and in severe cases cardiovascular compromise [2]. This trauma can be seen in many forms, including pneumothorax, subcutaneous emphysema, pneumomediastinum, pulmonary interstitial emphysema, air embolism, and pneumopericardium The pathophysiology behind these clinical diagnoses stems from the rupture of alveoli and the subsequent diffusion of air into these other spaces [2]. The air can diffuse into pulmonary capillaries, perivascular sheaths, and the pleural cavity itself [5]. Given this pathophysiology, it would seem patients with lung disease, such as asthma, chronic obstructive pulmonary disease, syncope, seizures, and panic disorders, would be at higher risk for pulmonary barotrauma in the right environment. Research has not shown this to be as conclusive as one would assume and further research with appropriate controls is needed [5]. In terms of diagnostic evaluation of patients with suspected barotrauma, plain radiography should be obtained at a minimum though further evaluation with CT can be indicated to assess for the location of trauma.

## Conclusions

In conclusion, this patient’s cause of mediastinal and subcutaneous emphysema likely was secondary to pulmonary barotrauma in the setting of breath holding while ascending from his repetitive dives. In this case, the patient was without any lung disease or other risk factors such as smoking to put him at increased risk. Chest radiography was able to diagnose the emphysema but ultimately CT was able to provide the extent of the pulmonary barotrauma and allow for extubation planning. This article provides a unique case report of severe barotrauma from mild depth dives without any use of underwater breathing apparatuses in an otherwise young and healthy patient, a presentation that could walk into any emergency department.
